# Traceability and dynamical resistance of precursor of extreme events

**DOI:** 10.1038/s41598-018-38372-y

**Published:** 2019-02-11

**Authors:** Thorsten Rings, Mahmood Mazarei, Amin Akhshi, Christian Geier, M. Reza Rahimi Tabar, Klaus Lehnertz

**Affiliations:** 1 Department of Epileptology, University of Bonn, Sigmund-Freud-Straße 25, 53105 Bonn, Germany; 2 Helmholtz-Institute for Radiation and Nuclear Physics, University of Bonn, Nussallee 14–16, 53115 Bonn, Germany; 3 Department of Physics, Sharif University of Technology, Tehran, 11155-9161 Iran; 4 Institute of Physics and ForWind, Carl von Ossietzky University of Oldenburg, Carl-von-Ossietzky-Straße 9–11, 26111 Oldenburg, Germany; 5 Interdisciplinary Center for Complex Systems, University of Bonn, Brühler Straße 7, 53175 Bonn, Germany

## Abstract

Extreme events occur in a variety of natural, technical, and societal systems and often have catastrophic consequences. Their low-probability, high-impact nature has recently triggered research into improving our understanding of generating mechanisms, providing early warnings as well as developing control strategies. For the latter to be effective, knowledge about dynamical resistance of a system prior to an extreme event is of utmost importance. Here we introduce a novel time-series-based and non-perturbative approach to efficiently monitor dynamical resistance and apply it to high-resolution observations of brain activities from 43 subjects with uncontrollable epileptic seizures. We gain surprising insights into pre-seizure dynamical resistance of brains that also provide important clues for success or failure of measures for seizure prevention. The novel resistance monitoring perspective advances our understanding of precursor dynamics in complex spatio-temporal systems with potential applications in refining control strategies.

## Introduction

Extreme events are usually considered as rare and unpredictable and/or as strongly deviating from normality and critically determine the evolution and character of a vulnerable human or natural system^1–6^. Earthquakes, tsunamis, or extreme weather events–such as heat waves, droughts, floods, heavy precipitation, or tornadoes–are well known extreme events and can lead to disasters when interacting with exposed or vulnerable systems. Likewise, events such as meltdown of nuclear power plants^7^, large-scale blackouts in power supply networks^8,9^, market crashes^10,11^, mass panics^12^, wars^13^, harmful algal blooms in marine ecosystems^14^, epileptic seizures in the human brain^15^, or even competition for attention in social media^16^ can have catastrophic consequences for the individual, society, finance, and nature.

Research into understanding mechanisms leading to extreme events in diverse systems saw a surge of activities over the past years^17–21^, resulting in a broad spectrum of methods aiming to identify precursors of extreme events^22–32^. Although this multitude of predictive methods indicates that truly generic warning signals are unlikely to exist^33^, the potential to identify precursors of extreme events offers a way forward–in spite of such seemingly unpredictable behavior–to develop strategies for adaptation, mitigation, and avoidance of such events. For such strategies to be effective, knowledge about the dynamical resistance of a system’s precursor is indispensable. Here, dynamical resistance refers to a system’s ability to adjust its activity to retain its basic functionality when internal or environmental changes occur^34^.

A continuous, data-driven monitoring of dynamical resistance remains an unsolved issue, especially in open and adaptive high-dimensional systems that are comprised of diverse sub-systems with different types of time-varying interaction, and whose dynamics is highly non-stationary. A prime example for such a system is the human brain, a complex network of highly interconnected networks, which are neither random nor entirely regular, and span multiple spatial scales (from individual cells and synapses via cortical columns to (sub)cortical areas). These networks support a rich repertoire of behavioral and cognitive functions, and in the case of brain pathologies, normal and abnormal functions and/or structures can coexist^35^.

We develop an approach that allows a time-resolved monitoring of dynamical resistance from multivariant observations of a system’s interaction dynamics only. Estimating resistance usually requires knowledge about a system’s different dynamical regimes and their response to some exogenous or endogenous perturbation^36,37^, an information which may not be accessible for the aforementioned systems. Here, we assume that induced and/or spontaneous changes in the system’s underlying coupling structure–as assessed by a time-series-based analysis of interactions–suffice to properly identify the system’s different dynamical regimes. An important estimate for dynamical resistance is the minimum “distance” between these operationally-defined regimes.

At the example of precursors of epileptic seizures in the human brain^32^, we demonstrate the potentials of our approach which eventually could be used to help maintain or move the epileptic brain and other vulnerable systems towards more desirable and sustainable dynamics, track thresholds of potential concern and help with evaluations on how such systems are being managed.

## Results

### A time-series-based approach to tracking dynamical resistance

**Figure 1:**
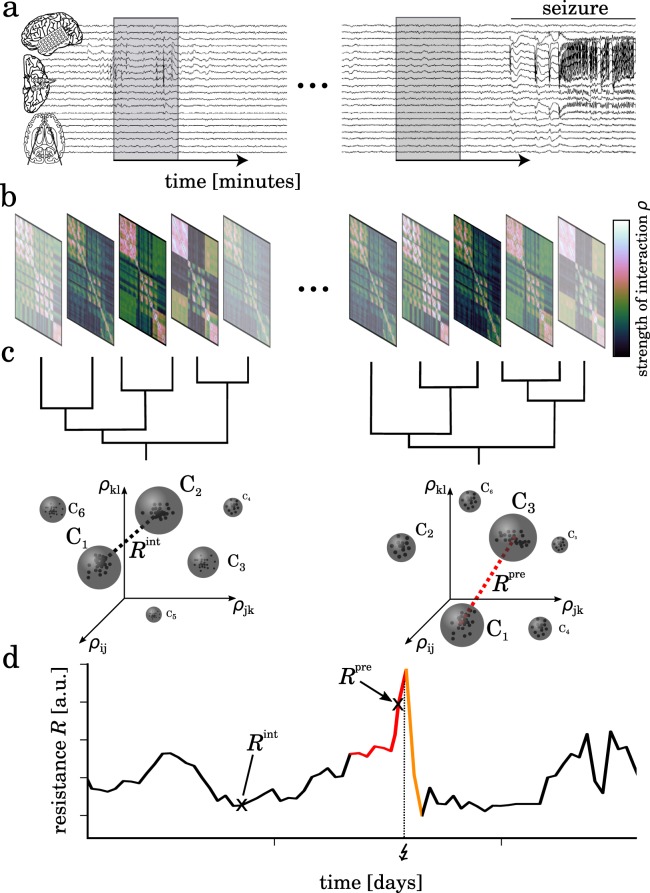
Tracking dynamical resistance of a system when transiting into and out of an extreme event. (**a**) The system’s dynamical coupling structure is probed from a sliding-window analysis of the strength of interactions between all pairs (i, j) of subsystems (here: phase-synchronization analysis of intracranial electroencephalographic signals from various brain regions). (**b**) Temporal sequence of symmetric interaction matrices *ρ*. (**c**) Similar coupling structures with small Euclidean separation in an abstract space spanned by all pairwise interactions *ρ*_ij_ are subsumed (hierarchical clustering analysis) to a finite number ${\mathscr{N}}$ of the system’s accessible dynamical regimes C_*n*_ (grey spheres). Black dots inside spheres indicate similar coupling structures at different, not necessarily consecutive times during a preset time window (here 1 h). Dynamical resistance *R* is defined as the minimum distance (dotted lines) between all accessible dynamical regimes. (**d)** Exemplary temporal evolution of dynamical resistance *R* when transiting into and out of an extreme event (here: epileptic seizure; onset marked with a bolt on x-axis). The inter-seizure period is colored black, the pre-seizure period (assumed duration 4 h) is colored red. The seizure and the post-seizure period (assumed duration 1.5 h) are colored orange. *R*^int^ denote resistance values from the inter-seizure period and *R*^pre^ those from the pre-seizure period.

Our approach (Fig. 1) is based on probing with high temporal resolution the dynamical coupling structure between interacting, even non-stationary and non-linear elements (or subsystems) of a system taking into account the subsystems’ individual signals only. We assess the dynamical coupling structure with an analysis of the strength of interactions between all pairs of subsystems (Methods), which results in a temporal sequence of interaction matrices *ρ*.

To illustrate the possible existence of various dynamical regimes, we define similarity between two matrices *ρ*(*t*_*k*_) and *ρ*(*t*_*l*_) at times *t*_*k*_ and *t*_*l*_ as $\xi ({t}_{k},\,{t}_{l})\equiv \Vert \rho ({t}_{k})-\rho ({t}_{l})\Vert$, where $\Vert \ldots \Vert$ denotes the Euclidean norm^38^. We emphasize that the similarity matrix *ξ* –estimated for all times *t*_*k*_ and *t*_*l*_ – contains pertinent information about the system’s dynamics, and that recurrent patterns in the similarity matrix indicate dynamical regimes^39^.

Now, in order to identify these regimes and their number we employ a time-resolved hierarchical clustering analysis of coupling structures in an abstract space spanned by all pairwise interactions (Methods). The minimum “distance” between the resulting clusters, that represent different dynamical regimes, is a central element of our approach and we take it as an estimate for dynamical resistance *R*: the larger this distance between regimes the higher is the capacity of a system to absorb disturbances and to reorganize while undergoing dynamical changes on the verge of an extreme event so as to still retain essentially the same functionality.

### Dynamical resistance of the human epileptic brain prior to seizures

We illustrate this new approach with an important example from clinical medicine. Epilepsy is a very common neurological disorder affecting approximately 65 million people worldwide^40^. It can not be controlled sufficiently with anti-epileptic drugs in approximately 30% of people with epilepsy^41^. Central to the burden of uncontrollable epilepsy for the person with epilepsy is the seemingly unpredictability of seizures. A novel approach to control previously uncontrollable seizures in people with epilepsy would consist in identifying seizure precursors combined with delivering a counteracting influence (e.g., neurostimulation, local cooling, local drug perfusion, or behavioral intervention)^42–44^ to prevent the generation of the extreme event^45,46^.

It is now well established that seizures in many people with epilepsy are preceded by a measurable changes in brain dynamics, which constitute a precursor of sufficient duration^32^. Lacking knowledge about the resistance of brain dynamics during such a precursor, it is unclear whether perturbing the brain with a counteracting influence would indeed prevent seizure generation. We address this problem and track dynamical resistance of brains of 43 subjects with epilepsy over days during which individual brains transit into and out of seizures (Methods).

**Figure 2:**
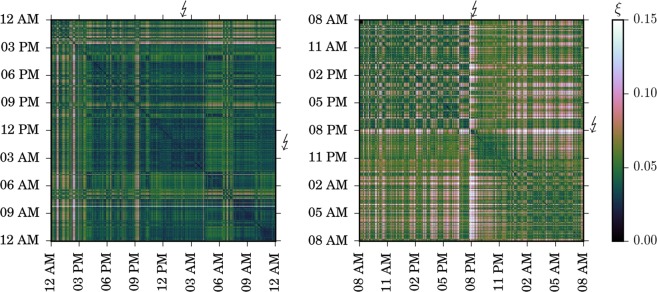
Changes of the brain’s dynamical coupling structure prior to and after epileptic seizures. Enlarged views of exemplary similarity matrices *ξ* covering a period of 24 h from two subjects with epilepsy. Seizure onsets are marked by bolts on top and to the right of each matrix. Seizures lasted for about 2 min, on average. For a given coupling structure–represented as a point on the matrix diagonal–, the similarity to coupling structures at previous times can be found on the vertical line above this point, or on the horizontal line to the left of this point. Dark shading (small values of *ξ*) denotes similar coupling structures and light shading (high values of *ξ*) denotes dissimilar coupling structures.

We study the individual brains’ dynamical coupling structures (Methods, Fig. 1) and derive a similarity matrix for each subject. Zooms into examples of such matrices from two subjects are shown in Fig. 2. This representation comprises changes of the coupling structure over 24 h in a single figure and allows to compare the similarity of coupling structures at different times. We find various recurrent patterns in each subject’s similarity matrix that suggests the presence of different dynamical regimes. Changes in the brains’ dynamical coupling structures occur on various time scales, and similarity to coupling structures at previous times appears to depend on the time of day. Prior to seizures, we observe time intervals of rather constant similarity that are rarely interrupted by changes in the coupling structure (Fig. 2, left panel) or intervals of comparably low similarity but with frequent intermissions (Fig. 2, right panel). We find similar changes in brains’ coupling structures preceding the other seizures. Nevertheless, we also observe similar patterning during time intervals far off seizure-related activities.

**Figure 3:**
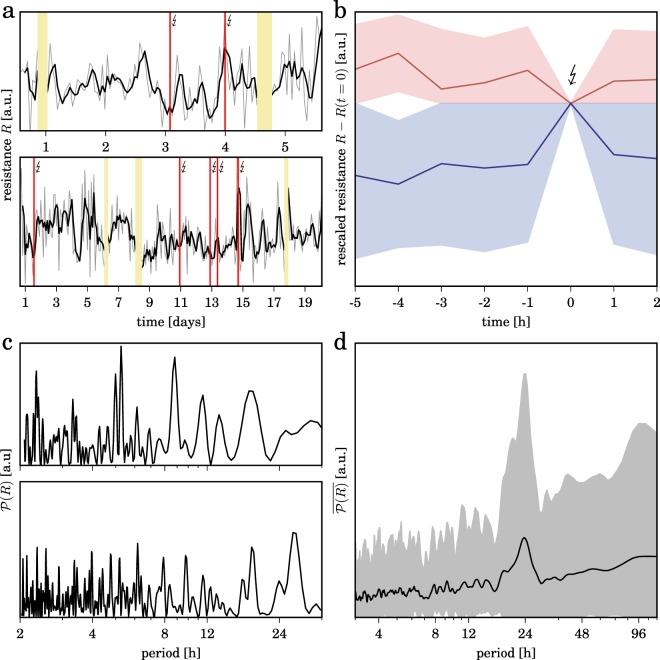
Time-dependent fluctuations of resistance of brain dynamics. (**a**) Temporal evolutions of dynamical resistance *R* (grey lines) from two subjects with epilepsy. Data are derived from ${\mathscr{N}}=6$ accessible dynamical regimes, and we find comparable time-dependent fluctuation with other number of accessible dynamical regimes (Supplementary Fig. S2). Smoothed temporal evolutions (moving average over 3 h) are shown as black lines; red vertical lines indicate times of seizure occurrence. Discontinuities in the temporal evolutions are due to recording gaps (colored khaki), and tics on x-axes denote midnight. (**b**) Temporal evolutions of dynamical resistance *R* when transiting into and out of epileptic seizures. Dynamical resistance values of the pre- and post-seizure period are rescaled to the value at seizure onset (*t* = 0). Decreasing values of resistance during the pre-seizure period (assumed duration: 4 h) are colored red and increasing values are colored blue. Means and standard deviations are shown by lines and shaded areas, respectively. (**c)** Periodograms^66^ ${\mathscr{P}}(R)$ of temporal evolutions of dynamical resistance depicted in (**a**) showing subject-specific ultradian (less than 24 h) and circadian (around 24 h) peaks in periodicity as well as infradian contributions (larger than 24 h)^67^. (**d)** Averaged periodogram $\overline{{\mathscr{P}}(R)}$ of temporal evolutions of dynamical resistance from all subjects showing a pronounced circadian peak in periodicity. Mean values and standard deviations are shown as solid lines and shaded areas, respectively.

Having identified different dynamical regimes for each subject (Methods), we find that dynamical resistance fluctuates over time to a lesser or greater extent (Fig. 3, panel a; Supplementary Fig. S1), however, with some peculiarities. First, in the hours prior to the vast majority of seizures (≈70%) we find dynamical resistance of brains to increase (Fig. 3, panel b). At first glance, this observation is quite surprising, since intuitively we would expect a diminished resistance of the epileptic brain in order to facilitate the generation of the extreme event seizure. Having said that, we here investigate seizures from people with drug-resistant epilepsies. We thus speculate that an increased dynamical resistance of the epileptic brain prior to seizures accounts for the reduced effectiveness of anticonvulsants.

**Figure 4:**
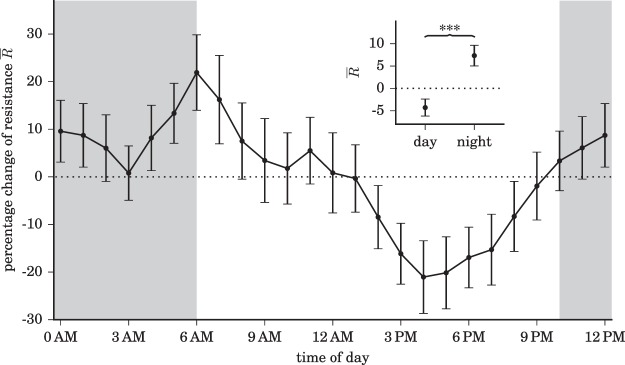
Dynamical resistance of brains depending on daytime. Hourly averaged dynamical resistance $\bar{R}$ (percent deviation from the 24 h average resistance; mean over data from all subjects with epilepsy; error bars represent standard error of the mean, s.e.m.). Data are derived from ${\mathscr{N}}=6$ accessible dynamical regimes, and we find comparable time-dependent fluctuation with other number of accessible dynamical regimes (Supplementary Fig. S3). Data are smoothed (moving average over 3 h), and lines are for eye guidance only. Inset shows means and s.e.m. of $\bar{R}$ values from recordings in the daytime and those at night-time (grey-shaded area). The black dotted lines indicate the mean resistance levels and asterisks indicate significant differences between the respective distributions (Kolmogorov-Smirnov test, *p* < 0.001).

Second, we observe some partly periodic temporal structure in the data (Fig. 3, panel c), which indicates dynamical resistance of brains to be influenced by ultradian, circadian, and probably even infradian rhythms (Fig. 3, panel d). When comparing resistance values from recordings in the daytime (6 am to 10 pm) with those at night-time (10 pm to 6 am; since no sleep-scoring was available for the patients investigated here, we cannot evaluate the influence of different sleep stages), we find the latter to be significantly increased (Fig. 4).

**Figure 5:**
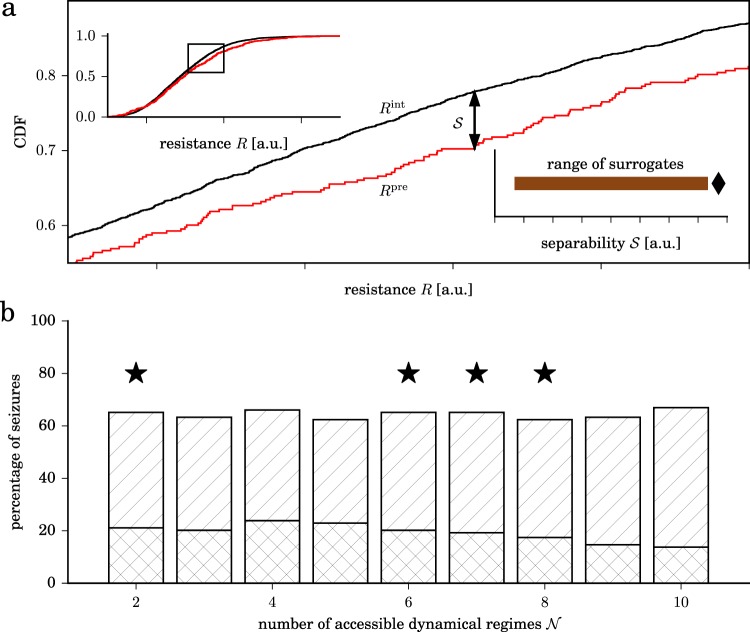
Dynamical resistance during pre-seizure periods and during inter-seizure periods is different. (**a**) Exemplary cumulative distribution functions (CDF) of resistance values from pre-seizure (*R*^pre^, red) and inter-seizure periods (*R*^int^, black). Maximum separability ${\mathscr{S}}$ of distributions (Kolmogorov-Smirnov statistic) is shown as double-headed arrow. The left inset shows the full CDF. The right inset shows separability ${\mathscr{S}}$ of distributions (diamond) to clearly exceed the range of ${\mathscr{S}}$ values (brown bar) derived from 19 seizure time surrogates (Methods). (**b**) Percentage of seizures with pre-seizure resistance values outside the range of values seen during inter-seizure periods for different numbers of accessible dynamical regimes (one-sigma range: hatched bars; two-sigma range: double-hatched bars). Stars mark number of accessible dynamical regimes for which separability for original seizure times exceeds the range of separability values derived from seizure time surrogates.

Although an influence of biological rhythms can to some extent be expected^47–50^, it is conceivable that the resulting waxing and waning of brain’s dynamical resistance but not a distinct seizure-generating mechanism leads to the observed pre-seizure changes. We therefore investigate whether fluctuations in brain’s dynamical resistance prior to seizures differ from the ones seen in between seizures. We borrow an approach commonly employed in seizure-prediction studies^22^ that is based on a surrogate-based comparison of the distributions of resistance values from the presumed pre-seizure periods and from inter-seizure periods (Methods and Fig. 5 panel a). We find that in more than two thirds of cases (68.8% of seizures; ${\mathscr{N}}=6$ accessible dynamical regimes), pre-seizure fluctuations in dynamical resistance clearly differ from those seen during inter-seizure periods (Fig. 5 panel b). We perform similar investigations on the level of individual brains and find pre-seizure fluctuations of dynamical resistance from all but one subjects to clearly differ from inter-seizure fluctuations (Supplementary Fig. S4). This makes our approach comparable to previous, high-performing^51^ precursor-indexing approaches.

We hypothesize that such abnormal pre-seizure fluctuations in dynamical resistance not only reflect the emergence of a seizure-permissive state but also indicate success/failure of potential seizure prevention strategies. We also hypothesize that tracking brain’s dynamical resistance over time provides important clues for targeted and personalized seizure prevention techniques, particularly about how and when to counteract. Both of these hypotheses are now testable by applying our approach to monitor dynamical resistance.

## Discussion

We have introduced a time-series-based approach to efficiently monitor dynamical resistance of complex systems prone to extreme events. Our approach differs from approaches that aim at quantifying stability. These typically require finding fixed points of a system for every possible value of control parameters. This ansatz, which is suitable for low-dimensional systems only^36^, is not applicable to complex systems that consist of a large number of coupled elements interacting with each other in a non-linear way on multiple scales. More recent developments^37^ require precise knowledge of the governing equations of motion. In contrast, our novel approach exploits–in a data-driven way–the system’s underlying dynamical coupling structure to define accessible dynamical regimes and derives an estimate for resistance from the minimum distance between regimes.

We have demonstrated the high suitability of our approach by a time-resolved tracking of dynamical resistance of individual brains, that transit into and out of extreme events *epileptic seizures*. The analysis goes substantially beyond state-of-the-art investigations of epileptic brain dynamics and also provides, for the first time, an objective measure for brain resistance, which is of paramount importance to optimize existing and to develop novel therapeutic interventions for controlling seizures.

Like any data-driven approach our method is limited by several assumptions: lacking an appropriate model for testing our approach’s general suitability, we introduced the method using a simple and robust measure for phase synchronization^52^ to quantify couplings, but the approach can to some extent also be implemented with other linear and non-linear quantifiers, for example using information-theoretic measures^53^. Phase-coherence analysis between all pairs of interacting subsystems assumes the dynamical coupling structure to mainly reflect phase-synchronization phenomena. As regards our brain analysis and despite there being strong evidence for phase synchronization to underlie various physiologic and pathophysiologic brain functions^54–56^, other forms of synchronization^57^ are certainly relevant for brain dynamics and discounting them can confound the assessment of resistance of brain dynamics. Likewise, there are other confounders that arise from the data recording^35^.

For the second major step of the method, namely the identification of dynamical regimes, we employed an hierarchical, centroid-based clustering algorithm. Our choice was based on the well-accepted notion that no clustering algorithm suitable to all problems and universally applicable to arbitrary datasets exists, and in view of our data analysis, we deliberately chose an easy-to-handle and widely-used algorithm. Since many of the existing algorithms–including the one employed here–fail to identify a large number of dynamical regimes from high-dimensional data due to the curse of dimensionality^58^, we restricted our downstream analyses to a lower number of accessible regimes. Given these limitations it is important to interpret dynamical resistance only relative to the variables that were taken into account.

A further complication are potentially predictive or other pertinent changes in dynamical resistance that are not captured with the temporal resolution considered here. Our hourly time resolution reflects a balance between a robust probing of the brain’s dynamical coupling structure based on non-stationary non-linear signals and an efficient centroidal Voronoi tessellation of the data space, thereby taking into account the duration of a potential precursor and the low-probability nature of epileptic seizures. Investigating dynamical resistance of other vulnerable systems may require an adaptation of the temporal resolution together with an allowance of a possible interplay of different time scales or time-varying time delays of couplings. More detailed research questions that aim at tracking dynamical resistance in prospective studies can take into account improvements towards real-time computations as well as adaptations of statistical approaches to judge sensitivity and specificity of critical changes in dynamical resistance.

Our novel resistance monitoring perspective provides a complementary approach to improve both detection and understanding of precursor dynamics in complex spatio-temporal systems, with potential applications in developing timely and targeted prevention techniques to facilitate better preparedness and to diminish loss of lives and damage to health, ecosystems, infrastructure, and property. We envisage advancements in various disciplines that are concerned with extreme events, ranging from the earth and climate sciences via the life and social sciences to finance and energy sciences.

## Materials and Methods

### Data

We analyse multiday, multichannel intracranial electroencephalographic (iEEG) signals from 43 subjects with epilepsy (25 female, 18 male, with ages between 9–65, average 34 years) that underwent presurgical evaluation for drug-resistant focal epilepsies. The data were part of previous studies^45,59^, and all subjects had signed informed consent that their clinical data might be used and published for research purposes. The study protocol had previously been approved by the ethics committee of the University of Bonn, and methods were carried out in accordance with the approved guidelines.

Signals were recorded from chronically implanted intrahippocampal depth electrodes and subdural grid- and/or strip-electrodes. Decisions regarding placement of electrodes were purely clinically driven and were made independently of this study. Signals were band-pass-filtered between 1–45 Hz, sampled at 200 Hz (sampling interval 5 ms) using a 16 bit analog-to-digital converter, and referenced against the average of signals of two electrode contacts outside the presumed focal region. Reference contacts were chosen individually for each subject. The recordings with, on average, 56 electrode contacts lasted between 18–258 h (average 107 h) during which 112 clinical seizures (3 seizures/subject, range 1–7) occurred. The time of seizure onset was visually identified on the iEEG as the time of earliest clear change from the subject’s baseline or normal background iEEG that eventually led to an electrographic seizure. We neglect subclinical seizures in our analyses.

### Probing the dynamical coupling structure

To quantify the strength of coupling between two sub-systems *X* and *Y*, we consider their output signals {*x*} and {*y*} each of length *T*. Since synchronization plays an important role in brain function and dysfunction, here we employ an analysis approach that is based on the concept of phase synchronization. We note that other analysis approaches like e.g. based on information theory provide similar results (Supplementary Figs S5 to S11).

We calculate in a time-resolved manner (non-overlapping windows of 20.48 s duration; *T* = 4096 data points) a phase-synchronization index (mean phase coherence)^52,60^ ${\rho }_{xy}=|\frac{1}{T}{\sum }_{t=1}^{T}\,{e}^{i({{\rm{\Phi }}}_{x}(t)-{{\rm{\Phi }}}_{y}(t))}|$ between phase time series {Φ_*x*_} and {Φ_*y*_} that we derive by Hilbert-transforming iEEG signals. As an estimator for the strength of interactions, *ρ*_*xy*_ takes on values between 0 and 1, indicating either complete asynchrony (zero coupling) or complete synchrony (strongest coupling) between sub-systems *X* and *Y*. We perform these steps of analysis for every possible combination of pairs of sampled brain regions (Fig. 1).

### Identifying dynamical regimes and estimating dynamical resistance

**Figure 6:**
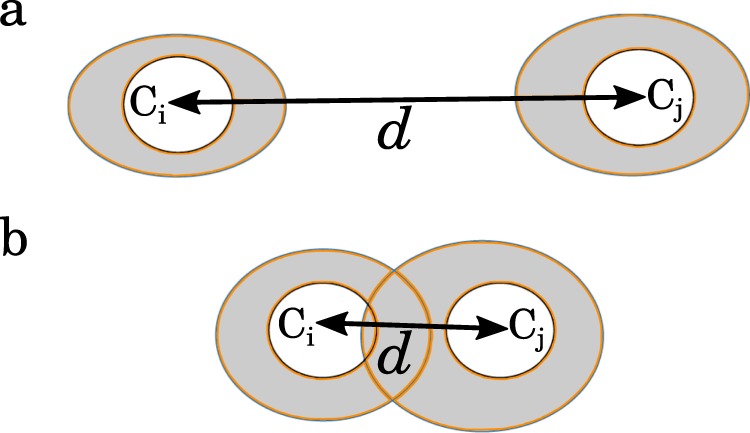
Geometrical illustration of dynamical resistance. Consider a system whose dynamical regimes C_*i*_ and C_*j*_ are separated in an abstract space by the minimum distance *d* (C_*i*_, C_*j*_). (**a)** If *d* is large, small endogenous and/or exogenous perturbations of the system that lead to small modifications of the system dynamics (grey-shaded areas) do not suffice to induce changes (e.g., a switch from regime C_*i*_ to C_*j*_) or a merging of different regimes: the system’s dynamics has a high resistance and can even absorb larger perturbations in the sense that dynamical regimes remain distinguishable. (**b)** With a small distance between dynamical regimes even tiny perturbations can induce changes or a merging of regimes; the system’s dynamics has a low resistance and can not absorb perturbations in the sense that dynamical regimes remain distinguishable.

We investigate changes in the similarity of coupling structures by means of a time-resolved clustering analysis (time base of 1 h; k-means algorithm^61^). With this technique, similar coupling structures from not necessarily consecutive points in time but with small Euclidean separation are subsumed to clusters in an abstract space spanned by all pairwise interactions. We identify these different clusters with different dynamical regimes (Fig. 1). The minimum distance *d* between regimes is an estimate for the dynamical resistance *R* of a system (Fig. 6): the larger this distance the higher is the system’s capacity to absorb disturbances, given that a larger distance between dynamical regimes requires a stronger perturbation to induce changes.

We initialize the clustering algorithm by assuming all coupling structures to belong to the same cluster. Next we divide this initial cluster into two clusters by randomly choosing two initial centroids of these sub-clusters. We then assign coupling structures to either the first or the second cluster according to a minimization of the variance of Euclidean separations of cluster constituents. We use the resulting centroids of these clusters as new initial centroids and repeat the process in an iterative manner^62^ until the current centroid equals the previous one. We minimize a possible bias due to the choice of the initial centroids by only considering those centroids for which we achieve a minimum variance of Euclidean separations of cluster constituents after reiterating the aforementioned steps of analysis for randomly chosen initial centroids.

The exact number of a system’s dynamical regimes is a priori unknown. We therefore repeat the clustering analysis for each of the two clusters separately in a divisive hierarchical manner^63^ until the Euclidean separation between all resulting clusters falls below numerically resolvable values. This approach allows us to identify up to ${\mathscr{N}}=10$ accessible dynamical regimes when applied to a temporal sequence of coupling matrices built from electroencephalographic signals and to create–for each analysis time frame–a dendrogram-like pattern of dynamical regimes^38^ that is sorted by the Euclidean separation between cluster centroids. The minimum Euclidean separation between cluster centroids is taken as the minimum distance *d* between dynamical regimes (Fig. 6).

### Testing for differences between dynamical resistances prior to and in between seizures

**Figure 7:**
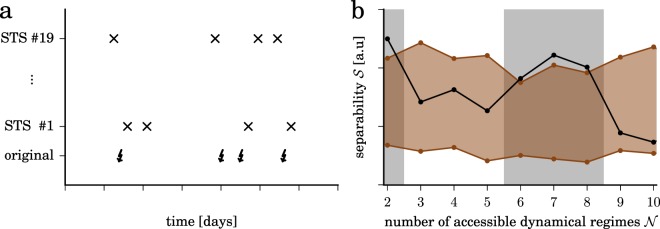
Evaluating separability of dynamical resistances from inter-seizure periods and pre-seizure periods with seizure time surrogates. (**a**) Exemplary sequence of original seizure times (lower trace, bolts) and surrogate times (upper traces, crosses). (**b**) Separability ${\mathscr{S}}$ of populations of resistance estimates from inter-seizure periods and pre-seizure periods for different numbers of accessible dynamical regimes. Separability for original seizure times is colored black, the range of separabilities derived with 19 seizure time surrogates is colored brown. Number of accessible dynamical regimes ${\mathscr{N}}$ for which separability for original seizure times passed the surrogate test are marked grey. Lines are for eye guidance only.

For our retrospective analyses, we use a statistical approach that is commonly employed in seizure-prediction studies^22^. In doing so, we assume existence of a critical transitional period (pre-seizure period) that is distinguishable from other, non-critical transitional periods that can be observed in between seizures (inter-seizure period). Given that seizure generation is likely to take place over minutes to hours^15^, we here assume the pre-seizure period to last for 4 h. For our analyses, we discard data from the 90 min interval after the onset of a seizure to minimize the risk of biasing our findings with effects related to brain dynamics during and after the seizure^64^. We then inspect separability ${\mathscr{S}}$ of populations of resistance estimates from the inter-seizure periods and from the pre-seizure periods, merging data from all subjects. To this end, we employ the Kolmogorov-Smirnov test to evaluate the difference between the two resistance populations, as it makes no assumption on the underlying distributions. We take the Kolmogorov-Smirnov statistic as an estimate for separability ${\mathscr{S}}$ and evaluate whether it deviates from randomness by testing against the null hypothesis of the non-existence of a critical transitional period. To do so, we employ the concept of seizure time surrogates (STS)^65^ that also allows us to account for possible confounding variables such as seizure clustering, daily rhythms, and changes in anticonvulsants. We derive STS from a random permutation of the original inter-seizure intervals and the time interval from the first seizure back to an arbitrarily defined starting point (Fig. 7 left). We reject the null hypothesis if separability for original seizure times exceeds the maximum one obtained with 19 STS (*p* < 0.05). This was the case for data derived with a number of accessible dynamical regimes ${\mathscr{N}}\in \{\mathrm{2,}\,\mathrm{6,}\,\mathrm{7,}\,8\}$ (Fig. 7 right). We present our findings obtained with ${\mathscr{N}}=6$; for the other numbers of accessible dynamical regimes, we obtained comparable findings.

## Supplementary information

Supplementary information

## References

[1] Bunde, A., Kropp, J. & Schellnhuber, H. -J. (eds) *The Science of Disaster* (Springer, Berlin, Heidelberg, 2002).

[2] Sornette, D. *Critical Phenomena in Natural Sciences* (Springer, Berlin, Heidelberg, 2003).

[3] Albeverio, S., Jentsch, V. & Kantz, H. (eds) *Extreme Events in Nature and Society*. The Frontiers Collection (Springer, Berlin, 2006).

[4] Field, C. B. *et al*. (eds) IPCC 2012: Managing the risks of extreme events and disasters to advance climate change adaptation. A special report of the intergovernmental panel on climate change (Cambridge University Press, Cambridge, UK, 2012).

[5] Helbing D (2013). Globally networked risks and how to respond. Nature.

[6] Buzulukova, N. *Extreme Events in Geospace: Origins, Predictability, and Consequences* (Elsevier, 2017).

[7] Christodouleas JP (2011). Short-term and long-term health risks of nuclear-power-plant accidents. New Engl. J. Med..

[8] Albert R, Albert I, Nakarado GL (2004). Structural vulnerability of the north american power grid. Phys. Rev. E.

[9] Buldyrev SV, Parshani R, Paul G, Stanley HE, Havlin S (2010). Catastrophic cascade of failures in interdependent networks. Nature.

[10] Feigenbaum JA (2001). A statistical analysis of log-periodic precursors to financial crashes. Quantitative Finance.

[11] Ivanov PC, Yuen A, Podobnik B, Lee Y (2004). Common scaling patterns in intertrade times of U. S. stocks. Phys. Rev. E.

[12] Helbing D (2001). Traffic and related self-driven many-particle systems. Rev. Mod. Phys..

[13] Hobsbawm, E. *The Age of Extremes: 1914–1991* (Abacus, London, 1994).

[14] Anderson DM, Cembella AD, Hallegraeff GM (2012). Progress in understanding harmful algal blooms: Paradigm shifts and new technologies for research, monitoring, and management. Annu. Rev. Mar. Sci..

[15] Lehnertz, K. *Epilepsy: extreme events in the human brain*, 123–143. In Albeverio *et al*.^3^ (2006).

[16] Miotto JM, Altmann EG (2014). Predictability of extreme events in social media. PLOS ONE.

[17] Sornette D, Ouillon G (2012). Dragon-kings: mechanisms, statistical methods and empirical evidence. Eur. Phys. J.-Spec. Top..

[18] Ansmann G, Karnatak R, Lehnertz K, Feudel U (2013). Extreme events in excitable systems and mechanisms of their generation. Phys. Rev. E.

[19] Adcock TAA, Taylor PH (2014). The physics of anomalous (rogue) ocean waves. Rep. Prog. Phys..

[20] Collins MJ (2014). Annual floods in New England (USA) and Atlantic Canada: synoptic climatology and generating mechanisms. Phys. Geogr..

[21] Mulhern C, Bialonski S, Kantz H (2015). Extreme events due to localization of energy. Phys. Rev. E.

[22] Mormann F, Andrzejak R, Elger CE, Lehnertz K (2007). Seizure prediction: the long and winding road. Brain.

[23] Manshour P (2009). Turbulencelike behavior of seismic time series. Phys. Rev. Lett..

[24] Scheffer M (2009). Early-warning signals for critical transitions. Nature.

[25] Scheffer M (2012). Anticipating critical transitions. Science.

[26] Boettiger C, Hastings A (2013). Tipping points: From patterns to predictions. Nature.

[27] Kuehn C, Zschaler G, Gross T (2015). Early warning signs for saddle-escape transitions in complex networks. Sci. Rep..

[28] Kuiper JJ (2015). Food-web stability signals critical transitions in temperate shallow lakes. Nat. Commun..

[29] Liu R, Chen P, Aihara K, Chen L (2015). Identifying early-warning signals of critical transitions with strong noise by dynamical network markers. Sci. Rep..

[30] Jurczyk J, Rehberg T, Eckrot A, Morgenstern I (2017). Measuring critical transitions in financial markets. Sci. Rep..

[31] Liang J, Hu Y, Chen G, Zhou T (2017). A universal indicator of critical state transitions in noisy complex networked systems. Sci. Rep..

[32] Kuhlmann L, Lehnertz K, Richardson MP, Schelter B, Zaveri HP (2018). Seizure prediction–ready for a new era. Nat. Rev. Neurol..

[33] Boettiger C, Ross N, Hastings A (2013). Early warning signals: the charted and uncharted territories. Theor. Ecol..

[34] Gao J, Barzel B, Barabási A-L (2016). Universal resilience patterns in complex networks. Nature.

[35] Lehnertz K, Geier C, Rings T, Stahn K (2017). Capturing time-varying brain dynamics. EPJ Nonlin. Biomed. Phys..

[36] May RM (1977). Thresholds and breakpoints in ecosystems with a multiplicity of stable states. Nature.

[37] Menck PJ, Heitzig J, Marwan N, Kurths J (2013). How basin stability complements the linear-stability paradigm. Nat. Phys..

[38] Münnix MC (2012). Identifying states of a financial market. Sci. Rep..

[39] Marwan N, Romano MC, Thiel M, Kurths J (2007). Recurrence plots for the analysis of complex systems. Phys. Rep..

[40] Moshé SL, Perucca E, Ryvlin P, Tomson T (2015). Epilepsy: new advances. Lancet.

[41] Kwan P, Schachter SC, Brodie MJ (2011). Drug-resistant epilepsy. N. Engl. J. Med..

[42] Fisher RS (2012). Therapeutic devices for epilepsy. Ann. Neurol..

[43] Ramgopal S (2014). Seizure detection, seizure prediction, and closed-loop warning systems in epilepsy. Epilepsy Behav..

[44] Michaelis R (2018). Psychological treatments for adults and children with epilepsy: Evidence-based recommendations by the International League Against Epilepsy Psychology Task Force. Epilepsia.

[45] Lehnertz K, Dickten H, Porz S, Helmstaedter C, Elger CE (2016). Predictability of uncontrollable multifocal seizures â€“ towards new treatment options. Sci. Rep..

[46] Kiral-Kornek I (2018). Epileptic seizure prediction using big data and deep learning: toward a mobile system. EBioMedicine.

[47] Hu K (2004). Endogenous circadian rhythm in an index of cardiac vulnerability independent of changes in behavior. Proc. Natl. Acad. Sci. USA.

[48] Ivanov PC, Hu K, Hilton MF, Shea SA, Stanley HE (2007). Endogenous circadian rhythm in human motor activity uncoupled from circadian influences on cardiac dynamics. Proc. Natl. Acad. Sci. USA.

[49] Parrino L, Vaudano AE (2018). The resilient brain and the guardians of sleep: New perspectives on old assumptions. Sleep Med. Rev..

[50] Khan S (2018). Circadian rhythm and epilepsy. Lancet Neurol..

[51] Gadhoumi K, Lina J-M, Mormann F, Gotman J (2016). Seizure prediction for therapeutic devices: A review. J. Neurosci. Methods.

[52] Mormann F, Lehnertz K, David P, Elger CE (2000). Mean phase coherence as a measure for phase synchronization and its application to the EEG of epilepsy patients. Physica D.

[53] Hlaváčková-Schindler K, Paluš M, Vejmelka M, Bhattacharya J (2007). Causality detection based on information-theoretic approaches in time series analysis. Phys. Rep..

[54] Engel AK, Fries P, Singer W (2001). Dynamic predictions: oscillations and synchrony in top–down processing. Nat. Rev. Neurosci..

[55] Lehnertz K (2009). Synchronization phenomena in human epileptic brain networks. J. Neurosci. Methods.

[56] Fell J, Axmacher N (2011). The role of phase synchronization in memory processes. Nat. Rev. Neurosci..

[57] Boccaletti S, Kurths J, Osipov G, Valladares DL, Zhou CS (2002). The synchronization of chaotic systems. Phys. Rep..

[58] Kriegel H-P, Kröger P, Zimek A (2009). Clustering high-dimensional data: A survey on subspace clustering, pattern-based clustering, and correlation clustering. ACM Trans Knowl Discov Data.

[59] Dickten H, Porz S, Elger CE, Lehnertz K (2016). Weighted and directed interactions in evolving large-scale epileptic brain networks. Sci. Rep..

[60] Porz S, Kiel M, Lehnertz K (2014). Can spurious indications for phase synchronization due to superimposed signals be avoided?. Chaos.

[61] MacQueen, J. B. Some methods for classification and analysis of multivariate observations. In Cam, M. L. & Neyman, J. (eds) *Proceedings of 5th Berkeley Symposium on Mathematical Statistics and Probability*, 281–297 (University of California Press, Berkeley, USA, 1967).

[62] Lloyd S (1982). Least squares quantization in PCM. IEEE Trans. Inf. Theor..

[63] Rokach, L. & Maimon, O. Clustering methods. In Maimon, O. & Rokach, L. (eds.) *Data mining and knowledge discovery handbook*, 321–352 (Springer, Boston, MA, 2005).

[64] So NK, Blume WT (2010). The postictal EEG. Epilepsy Behav..

[65] Andrzejak RG (2003). Testing the null hypothesis of the nonexistence of a preseizure state. Phys. Rev. E.

[66] Press WH, Rybicki GB (1989). Fast algorithm for spectral analysis of unevenly sampled data. Astrophys. J..

[67] Baud MO (2018). Multi-day rhythms modulate seizure risk in epilepsy. Nat. Commun..

