# Supplementary material for: Traceability and dynamical resistance of precursor of extreme events
Source: Sci Rep. 2019 Feb 11;9:1744. doi: 10.1038/s41598-018-38372-y (PMC6370838; doi:10.1038/s41598-018-38372-y)
Supplement: Supplementary file 1 — Supplementary information [file 41598_2018_38372_MOESM1_ESM.pdf]

Supplementary information for "Traceability and dynamical resistance of precursor of extreme events"

Thorsten Rings[1,2], Mahmood Mazarei[3], Amin Akhshi[3],
Christian Geier[1,2], M. Reza Rahimi Tabar[3,4], Klaus Lehnertz[1,2,5,*]

[1]Department of Epileptology, University of Bonn,
Sigmund-Freud-Straße 25, 53105 Bonn, Germany

[2]Helmholtz-Institute for Radiation and Nuclear Physics,
University of Bonn, Nussallee 14–16, 53115 Bonn, Germany

[3]Department of Physics, Sharif University of Technology,
Tehran 11155-9161, Iran

[4]Institute of Physics and ForWind, Carl von Ossietzky University of Oldenburg,
Carl-von-Ossietzky-Straße 9–11, 26111 Oldenburg, Germany

[5]Interdisciplinary Center for Complex Systems, University of Bonn,
Brühler Straße 7, 53175 Bonn, Germany

[*]To whom correspondence should be addressed; E-mail: klaus.lehnertz@ukbonnde.

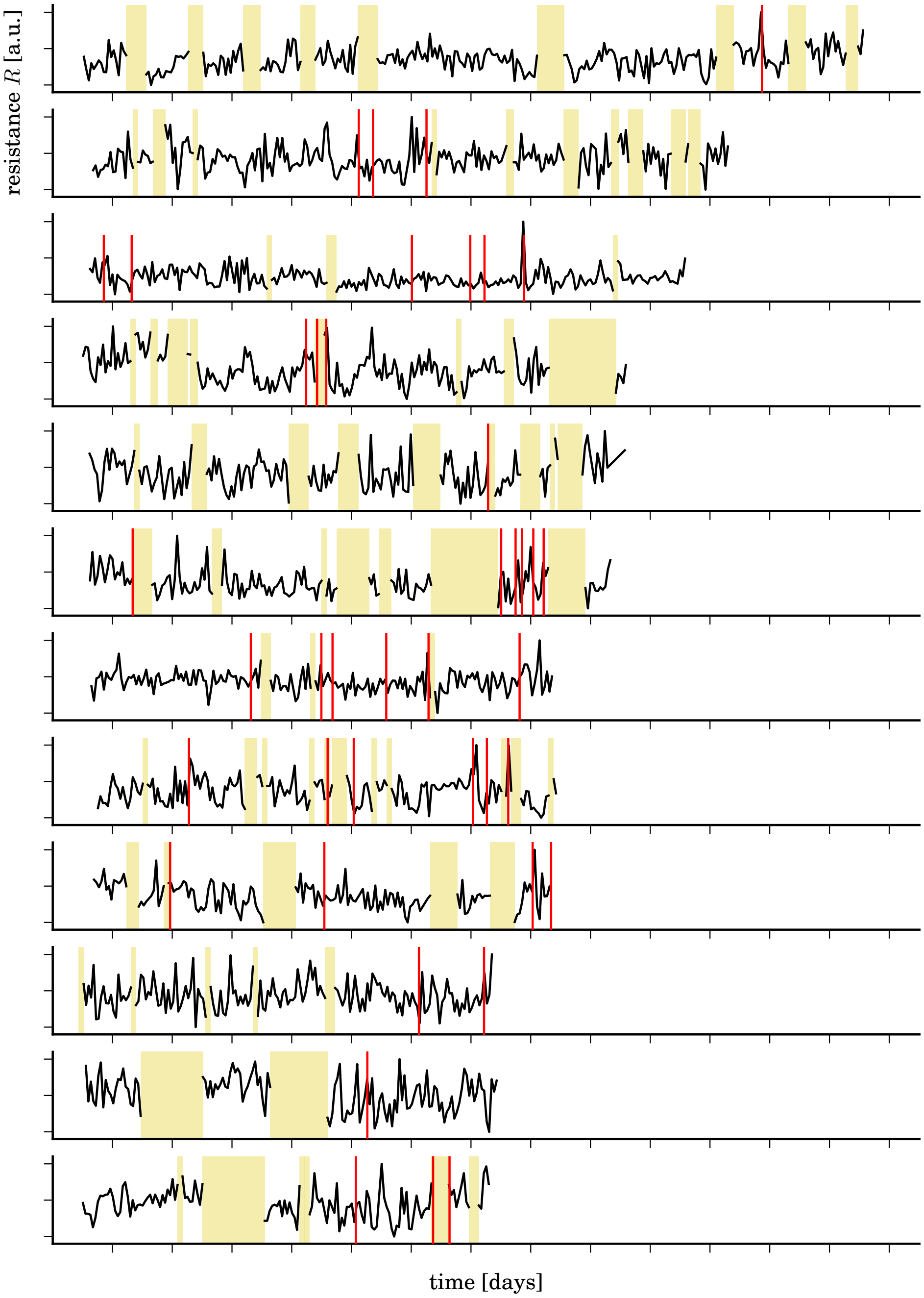

Figure S1: **Time-dependent fluctuations of brain dynamical resistance from all subjects.** Smoothed temporal evolutions of dynamical resistance R (moving average over 3 h) from $\mathcal{N} = 6$ accessible dynamical regimes. Red vertical lines indicate times of seizure occurrence. Discontinuities in the temporal evolutions are due to recording gaps (colored khaki), and tics on x-axes denote midnight. Data sorted by duration of recording; continued on next pages.

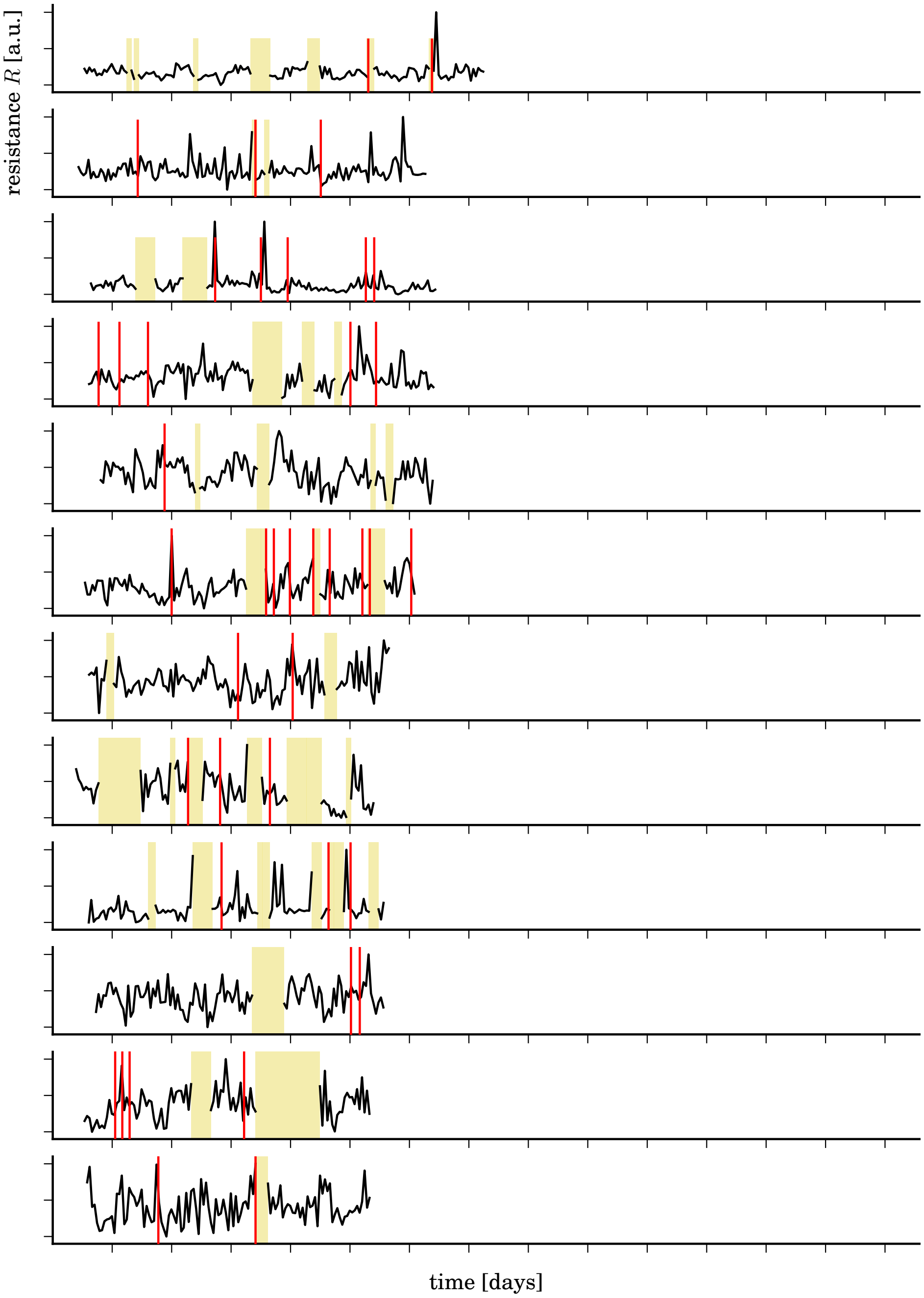

Continuation of Fig. S1.

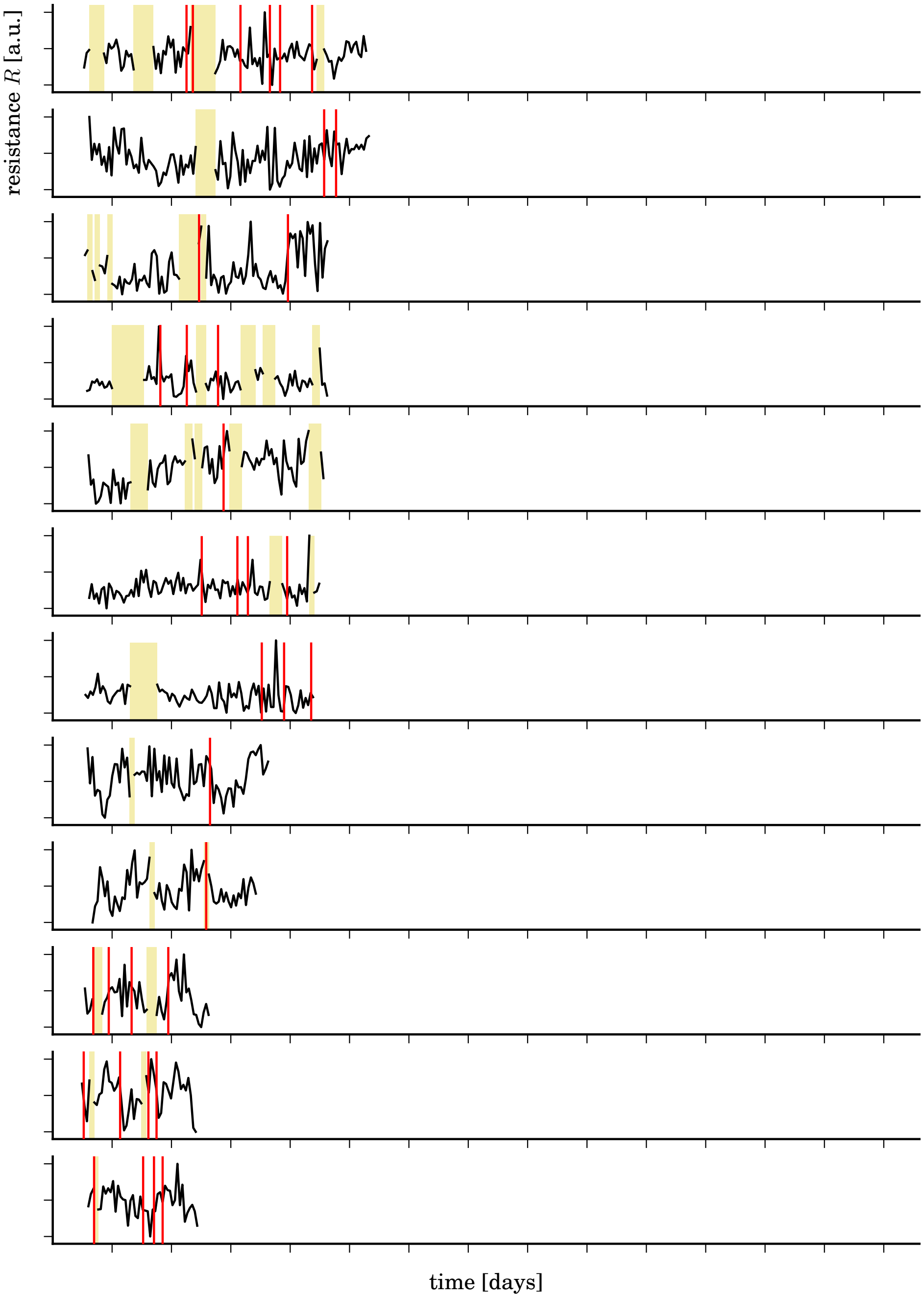

Continuation of Fig. S1.

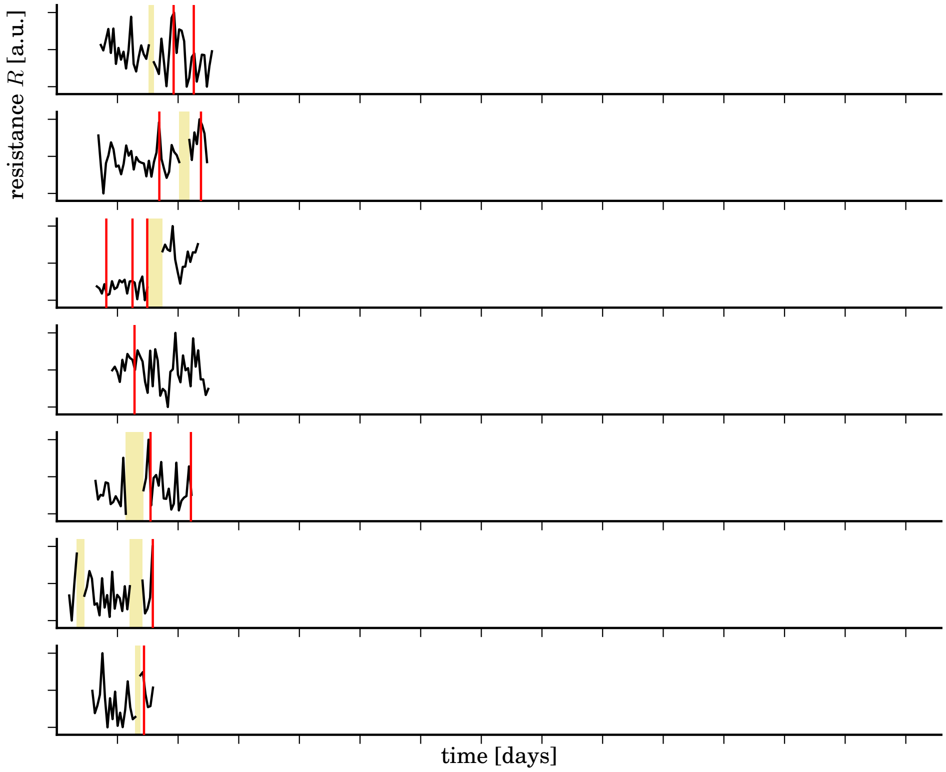

Continuation of Fig. S1.

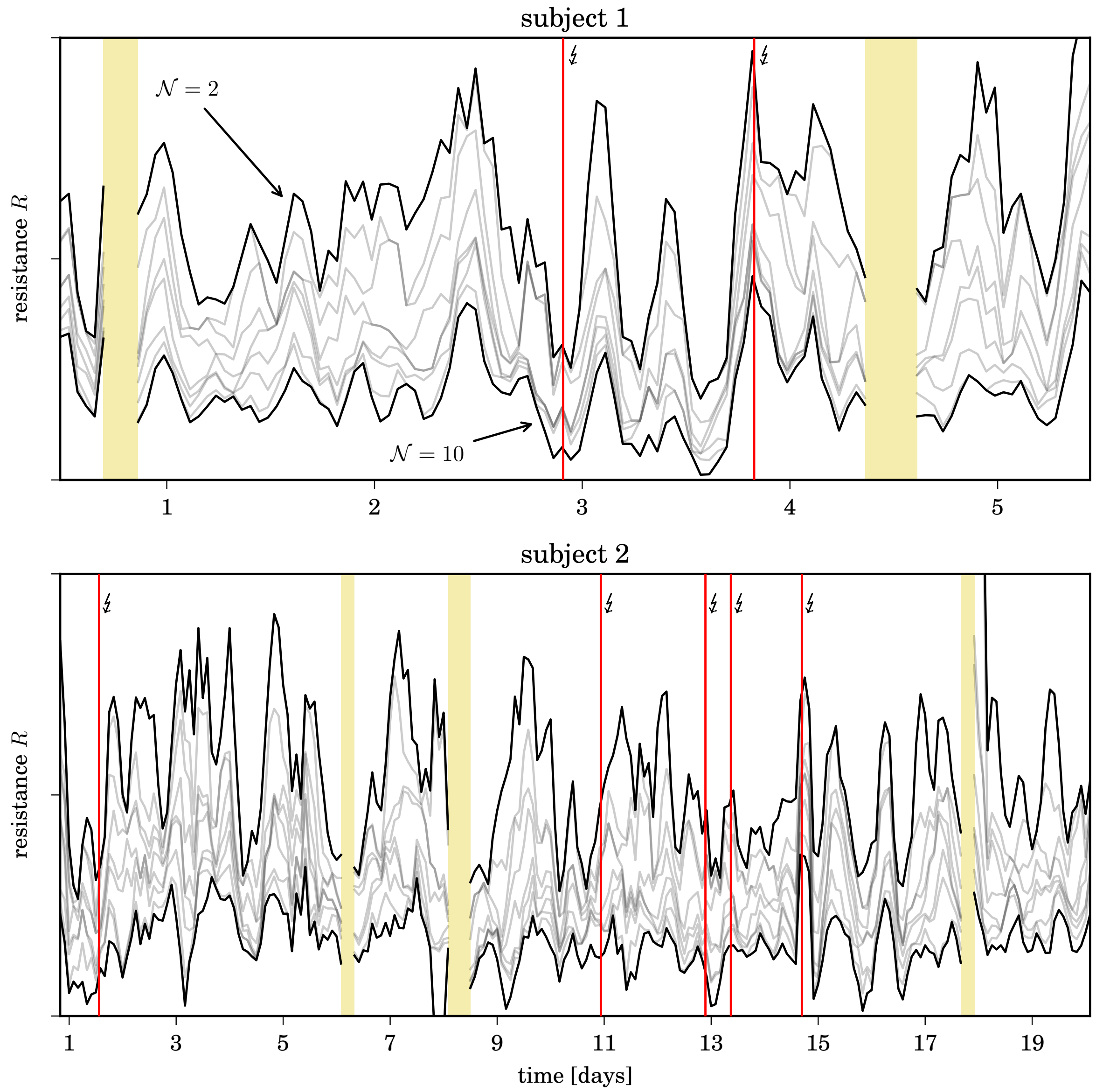

Figure S2: **Time-dependent fluctuations of brain dynamical resistance.** Smoothed temporal evolutions (moving average over 3 h) of dynamical resistance R from two subjects with epilepsy (see Fig. 3a in main text) for different numbers $\mathcal{N}$ of accessible dynamical regimes. Red vertical lines indicate times of seizure occurrence. Discontinuities in the temporal evolutions are due to recording gaps (colored khaki), and tics on x-axes denote midnight.

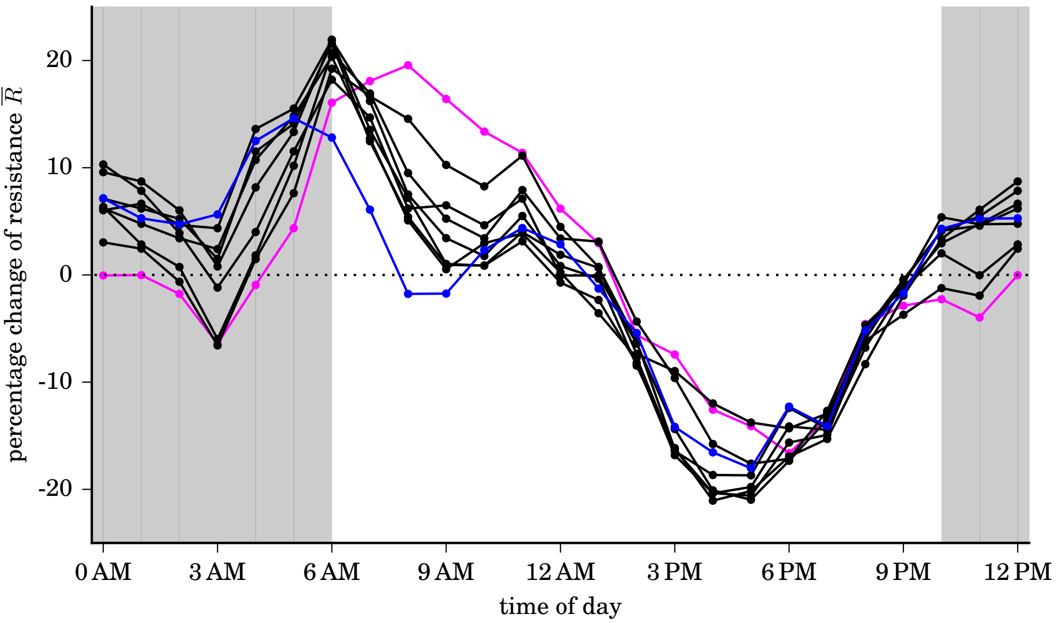

Figure S3: **Dynamical resistance of brains depending on daytime.** Hourly averaged dynamical resistance $\overline{R}$ (percent deviation from the 24 h average resistance; mean over data from all subjects with epilepsy; see Fig. 4 in main text) for different numbers $\mathcal{N}$ of dynamical regimes ($\mathcal{N} = 2$ is shown in magenta, $\mathcal{N} = 10$ is shown in blue). The grey-shaded area marks night times and the black dotted line indicates the mean resistance level.

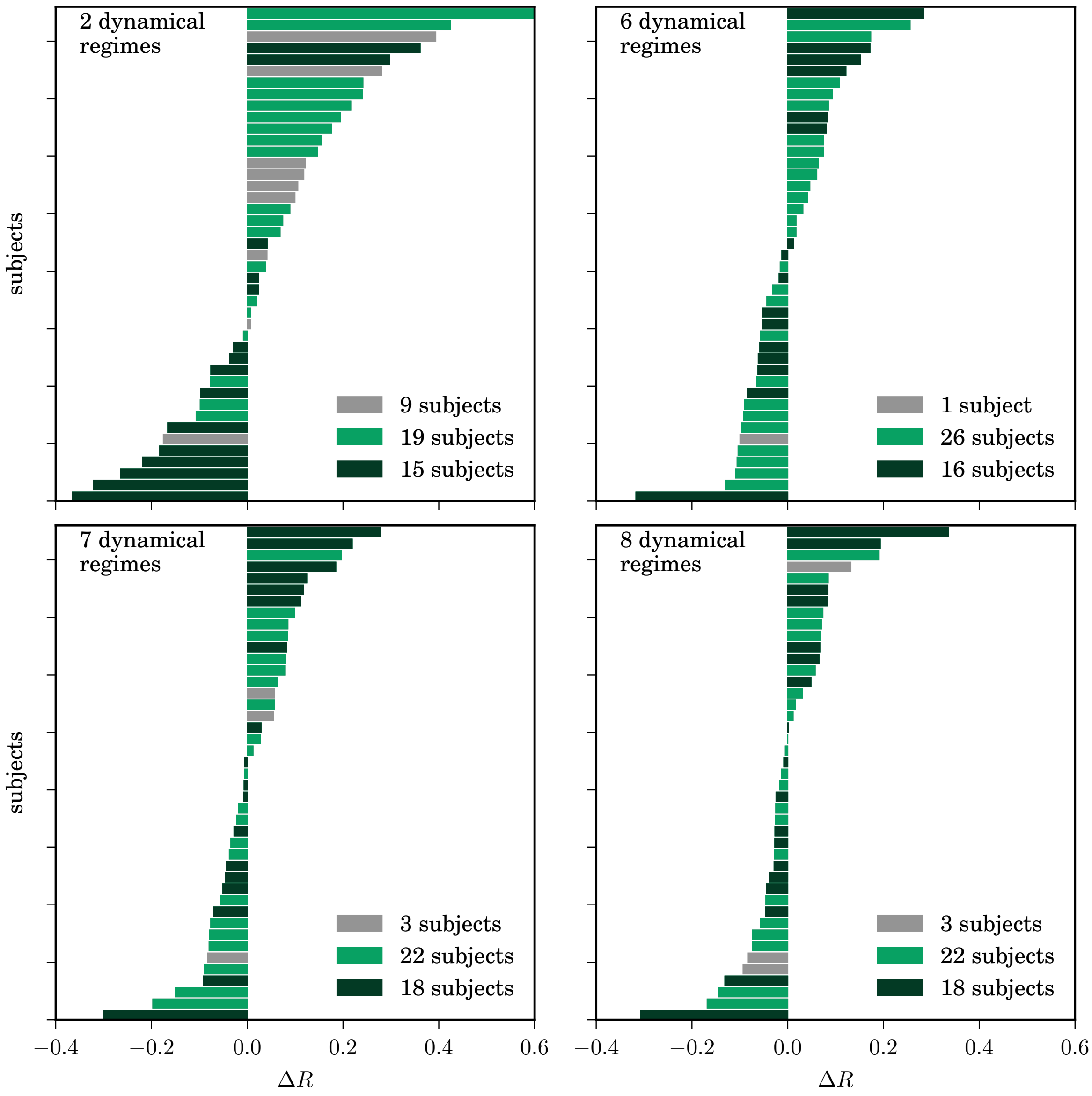

Figure S4: **Dynamical resistance of individual brains prior to epileptic seizures.** Relative deviation $\Delta R = \left(\overline{R^{\text{pre}}} - \overline{R^{\text{int}}}\right) / \overline{R^{\text{int}}}$ of mean resistance values from pre-seizure periods ($\overline{R^{\text{pre}}}$) from those from inter-seizure periods ($\overline{R^{\text{int}}}$) for each subject with epilepsy and for different numbers of dynamical regimes $\mathcal{N} \in \{2, 6, 7, 8\}$. $\Delta R > 0$ ($\Delta R < 0$) indicates higher (lower) dynamical resistance during pre-seizure periods. The coloring of each subject's bar marks the efficiency of our resistance-monitoring approach to index precursory states: pre-seizure fluctuations outside the one-sigma (two-sigma) range of inter-seizure fluctuations are colored light green (dark green), pre-seizure fluctuations inside the distribution of inter-seizure fluctuations are colored grey.

Probing the dynamical coupling structure with an information-theory-based quantifier.

As an alternative approach to probe the dynamical coupling structure (*1, 2*), we used the order parameter γ (*3*) to quantify the strength of coupling between two sub-systems X and Y. This approach requires symbol time series $\{\hat{x}\}$ and $\{\hat{y}\}$ that we derived from the iEEG time series $\{x\}$ and $\{y\}$, each of length T, using a technique of symbolization, which is based on a permutation of the amplitude values of a time series (*4*). We denote with l and m the embedding delay and the embedding dimension, which have to be chosen appropriately for symbolization (e.g., by making use of embedding theorems and by choosing l in the order of the first zero-crossing of the autocorrelation function to achieve (at least linearly) independent state-space vectors). Then m amplitude values $s_j := \left(x_j, x_{j+l}, \ldots, x_{j+l(m-j)}\right)$ for a given, but arbitrary time index j are arranged in ascending order $x_{j+l(k_{j1}-1)} \leq x_{j+l(k_{j2}-1)} \leq \cdots \leq x_{j+l(k_{jm}-1)}$ with rank k_{ji} and $i \in \{1, \ldots, m\}$. Equal amplitude values are arranged by their time index such that $k_{j1} < k_{j2}$ if $x_{j+l(k_{j1}-1)} = x_{j+l(k_{j2}-1)}$. This ensures that every s_j is uniquely mapped onto one of the $m!$ possible permutations. A permutation symbol is defined as $\hat{x}_j := (k_{j1}, k_{j2}, \ldots, k_{jm})$, and relative frequencies of symbols are estimates for (joint and conditional) probabilities of the sequences of permutation indices.

In order to derive an estimator for the strength of an interaction, we assess the consistency of changing tendencies of temporal permutation entropies $H_X = -\sum_j p(\hat{x}_j) \log p(\hat{x}_j)$ of symbol time series by splitting the time series into $\eta = 1, \ldots, N_\eta$ overlapping windows w_η with N_w data points each. The tendency can be quantified with the coefficient $S_X(w_\eta)$, which attains a value of 1 if $H_X(w_{\eta+1}) > H_X(w_\eta)$, and -1 otherwise. The permutation entropies $H_Y(w_\eta)$ and the coefficient $S_Y(w_\eta)$ are defined in complete analogy. The in-step behavior of H_X and H_Y

will be identical if systems X and Y are fully coupled, and the order parameter γ reads:

$$\gamma = \frac{1}{N_\eta} \sum_{\eta=1}^{N_\eta} S_X(w_\eta) S_Y(w_\eta).$$

γ will be close to unity for fully coupled systems X and Y and zero for independent ones. Note that γ might attain slightly negative values. γ increases monotonically with an increasing coupling strength and thus serves as an estimator for the strength of an interaction. Following previous work (*5*), we chose $m = 5$ and $l = 3$ for symbolization and estimated γ in a time-resolved manner (non-overlapping windows of 20.48 s duration; $T = 4096$ data points) with $N_w = 2048$ and $N_\eta = 204$.

This approach to probing the dynamical coupling structure allows us to identify up to $\mathcal{N} = 10$ accessible dynamical regimes when applied to a temporal sequence of coupling matrices built from electroencephalographic signals. The following Figs. S5 to S11 summarize our main findings obtained with the information-theory-based quantifier.

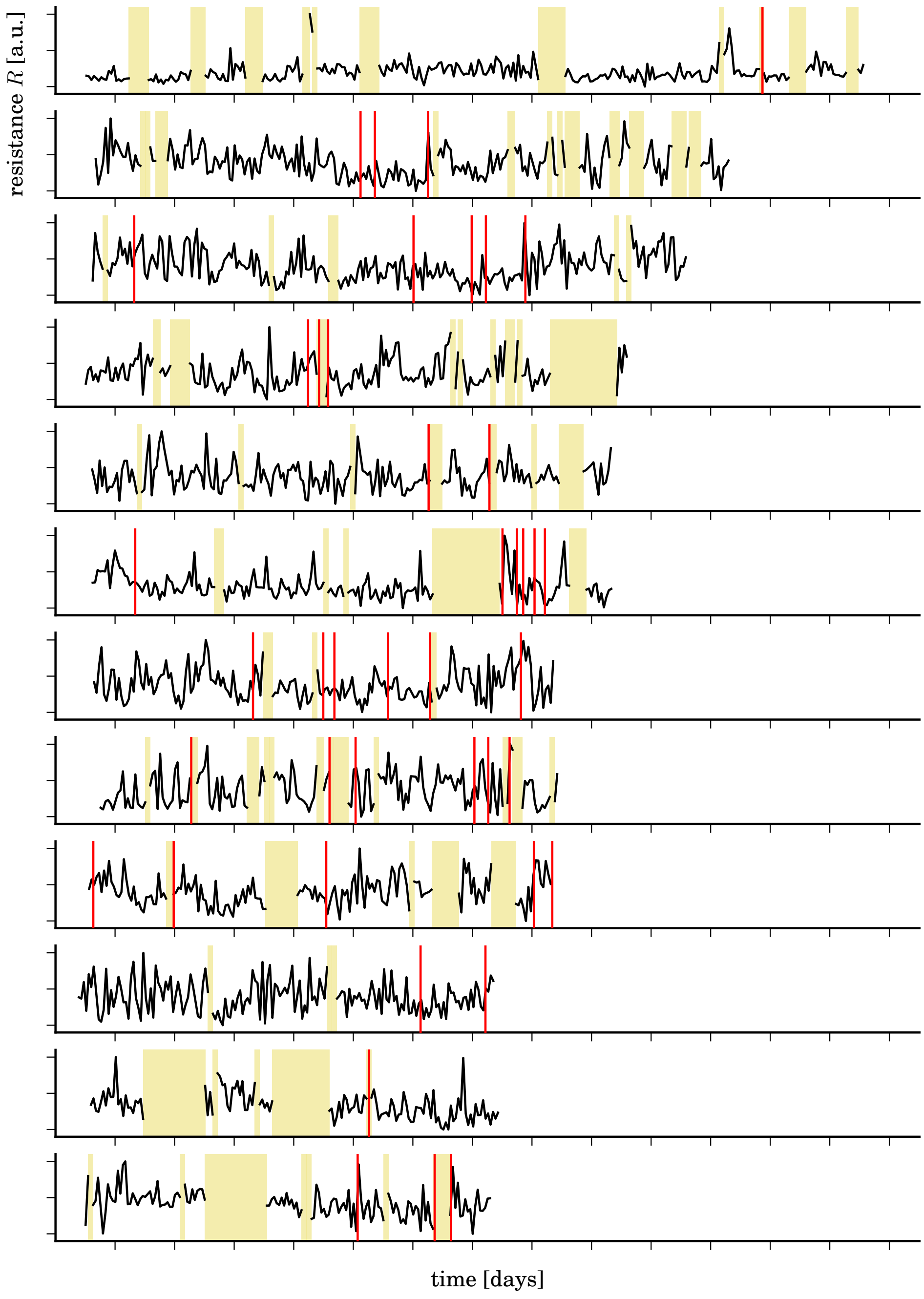

Figure S5: Same as Fig. S1, but findings derived with the information-theory-based quantifier (γ) of the dynamical coupling structure. Data sorted by duration of recording; continued on next pages.

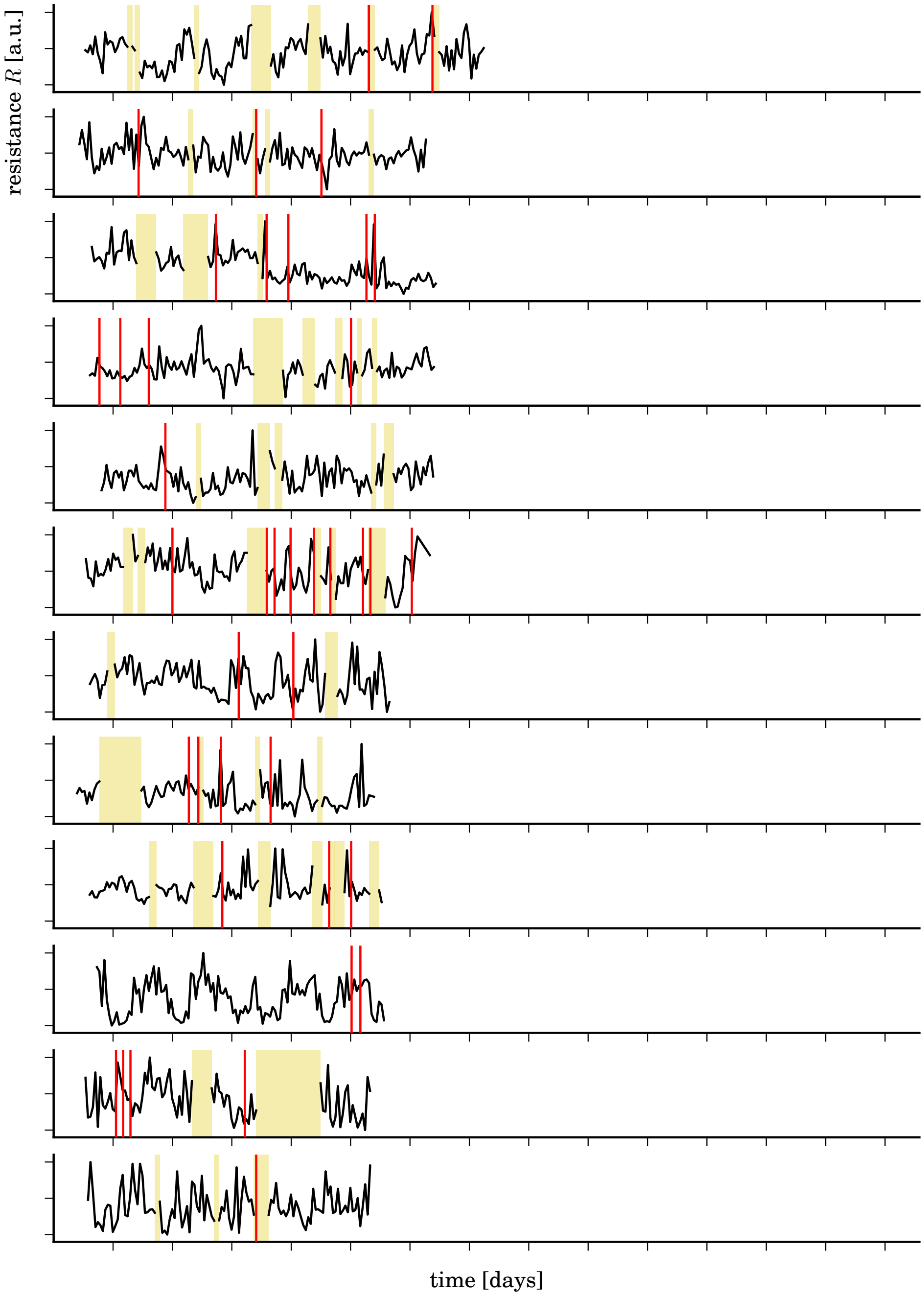

Continuation of Fig. S5.

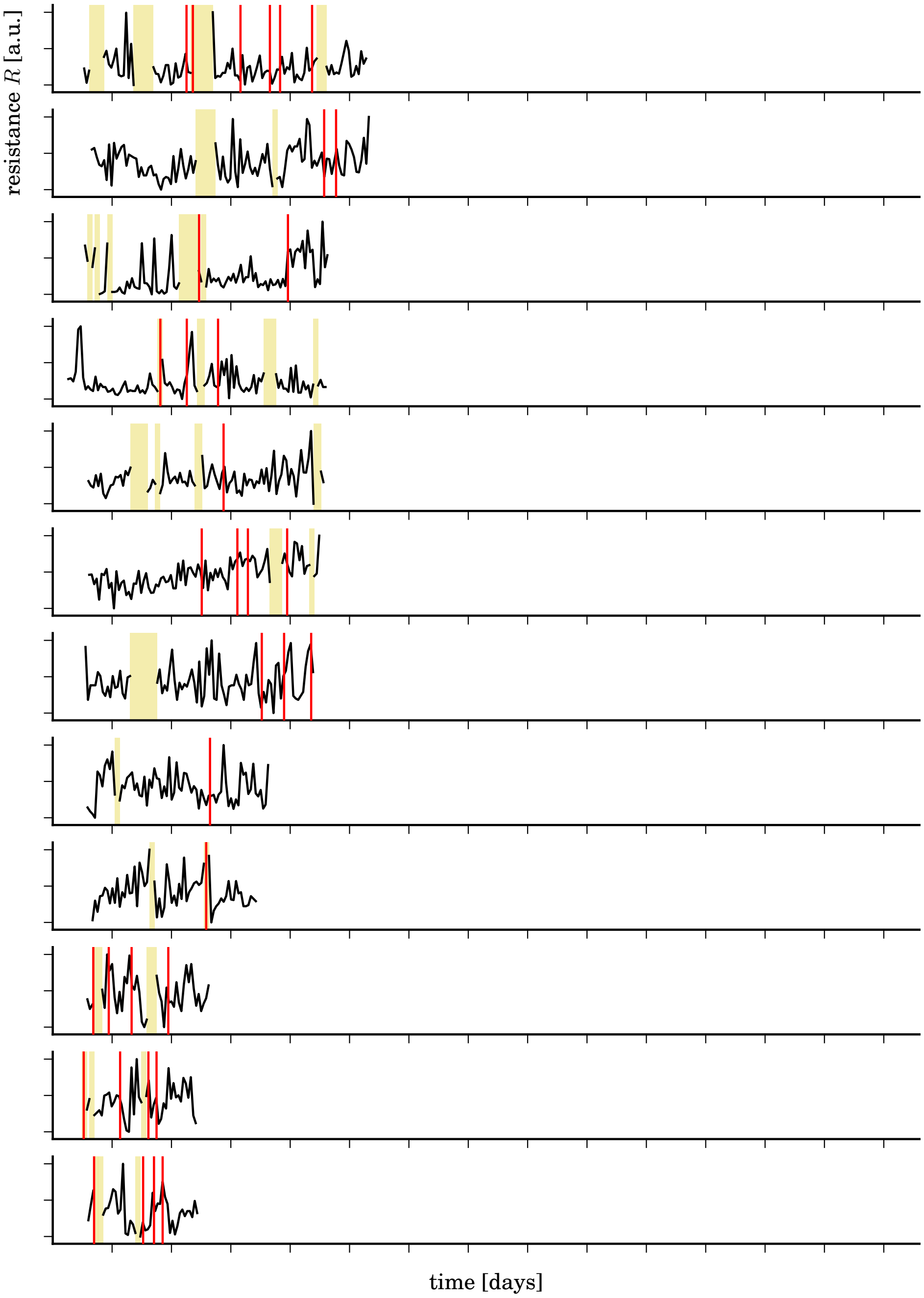

Continuation of Fig. S5.

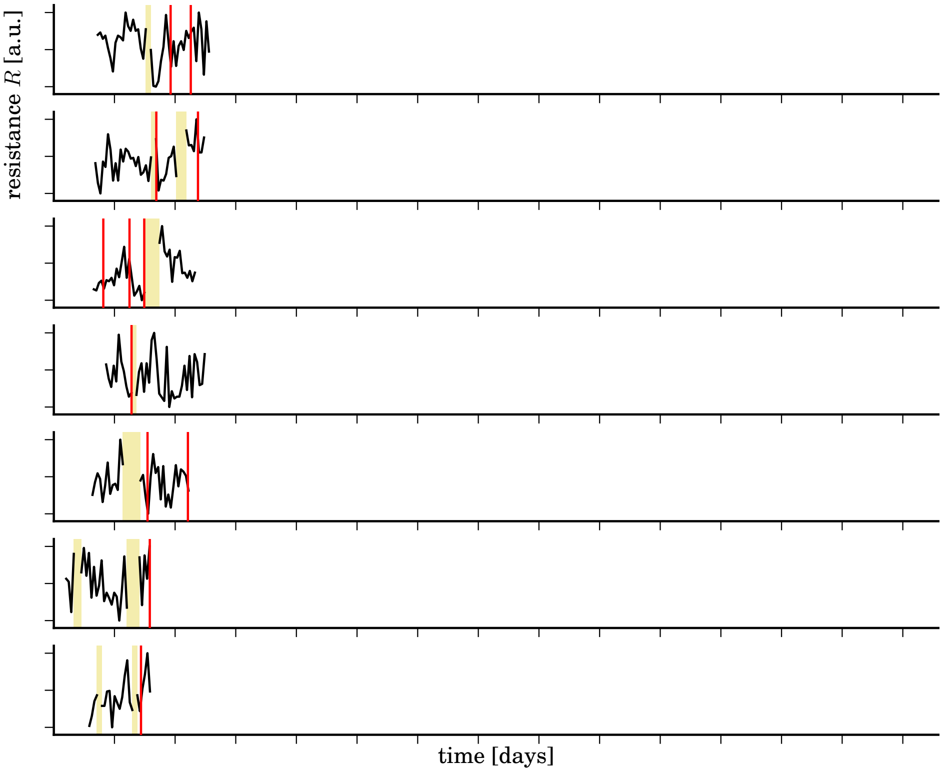

Continuation of Fig. S5.

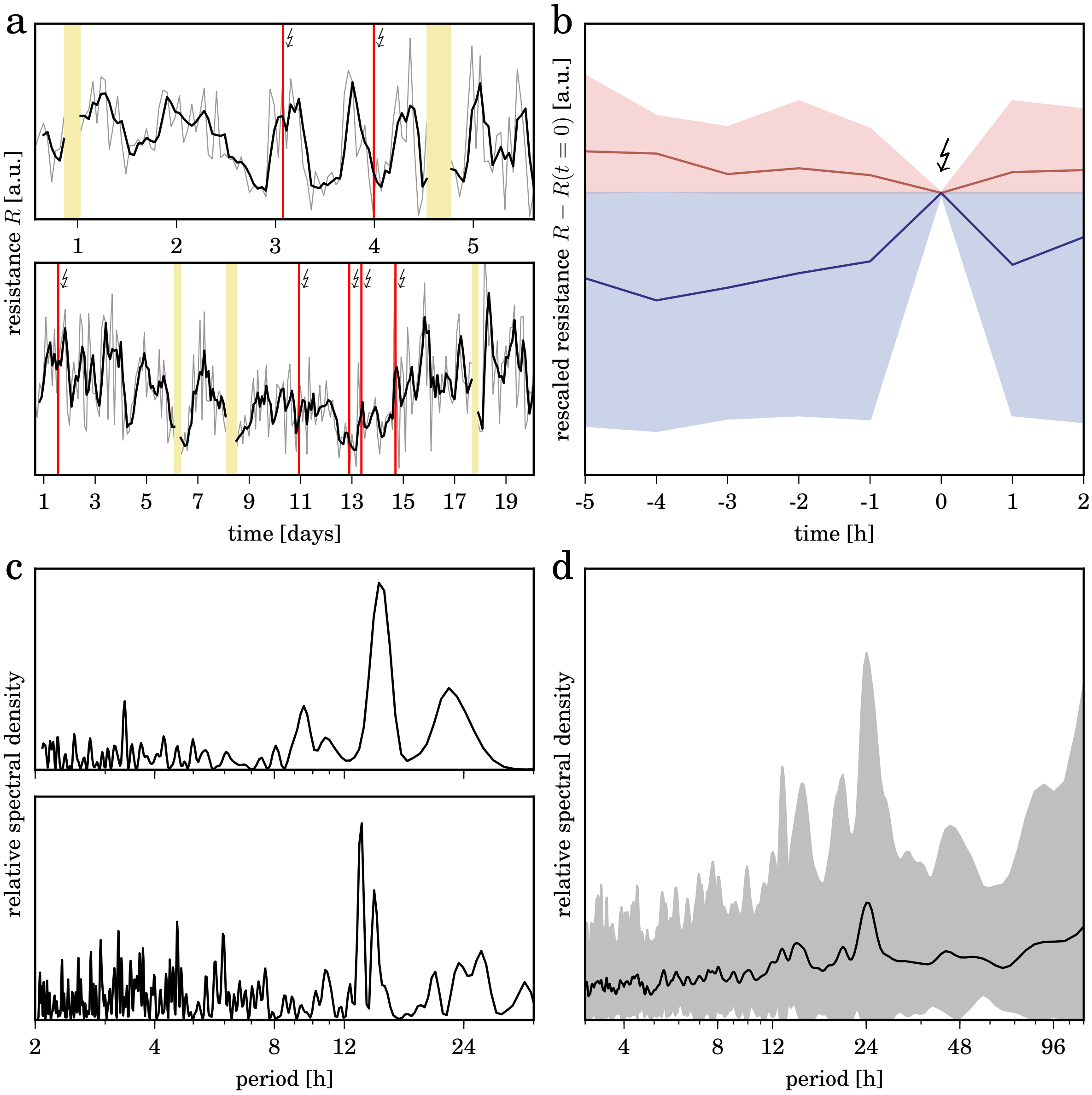

Figure S6: Same as Fig. 3 (main text), but findings derived with the information-theory-based quantifier (γ) of the dynamical coupling structure.

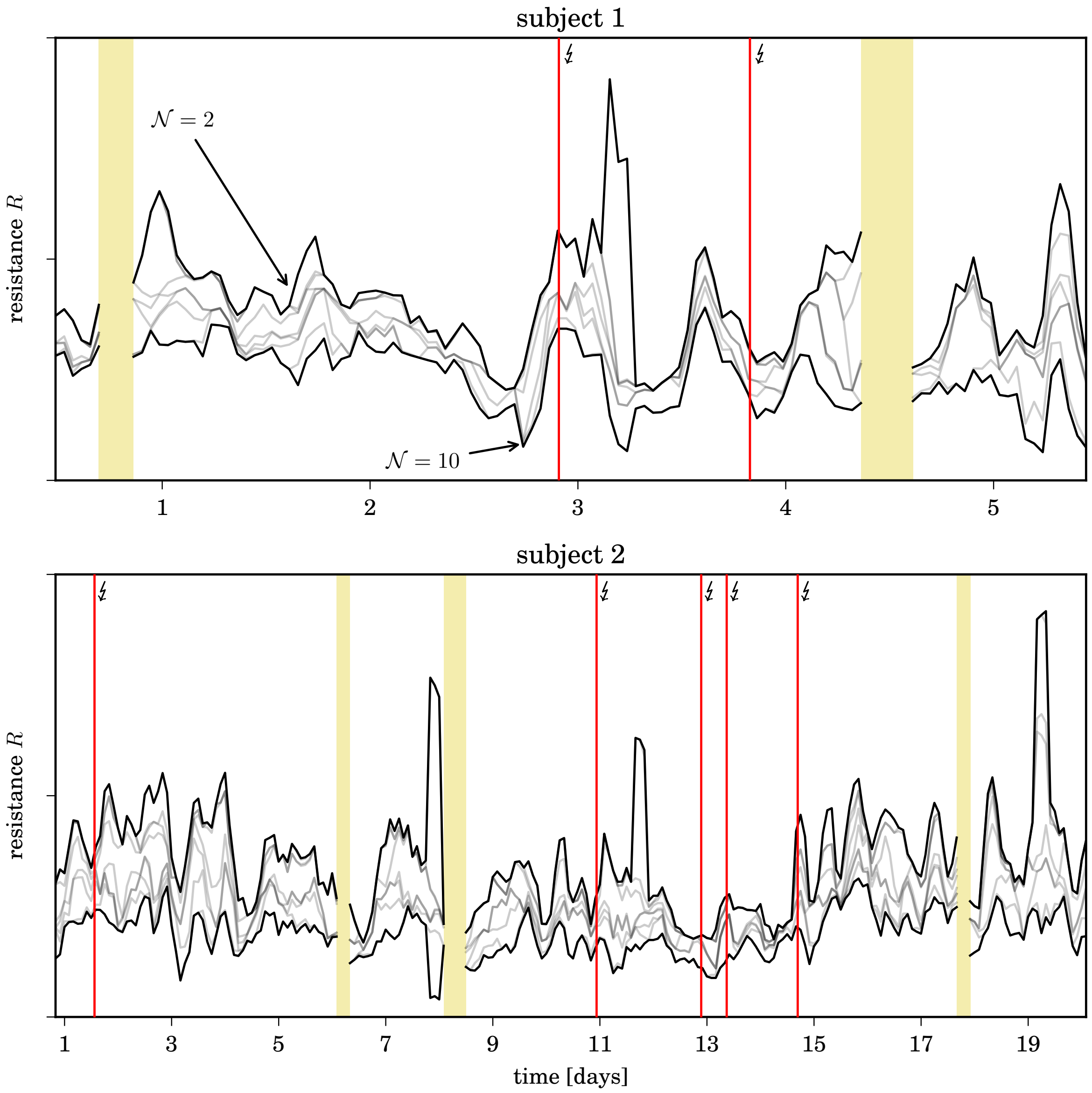

Figure S7: Same as Fig.S2, but findings derived with the information-theory-based quantifier (γ) of the dynamical coupling structure.

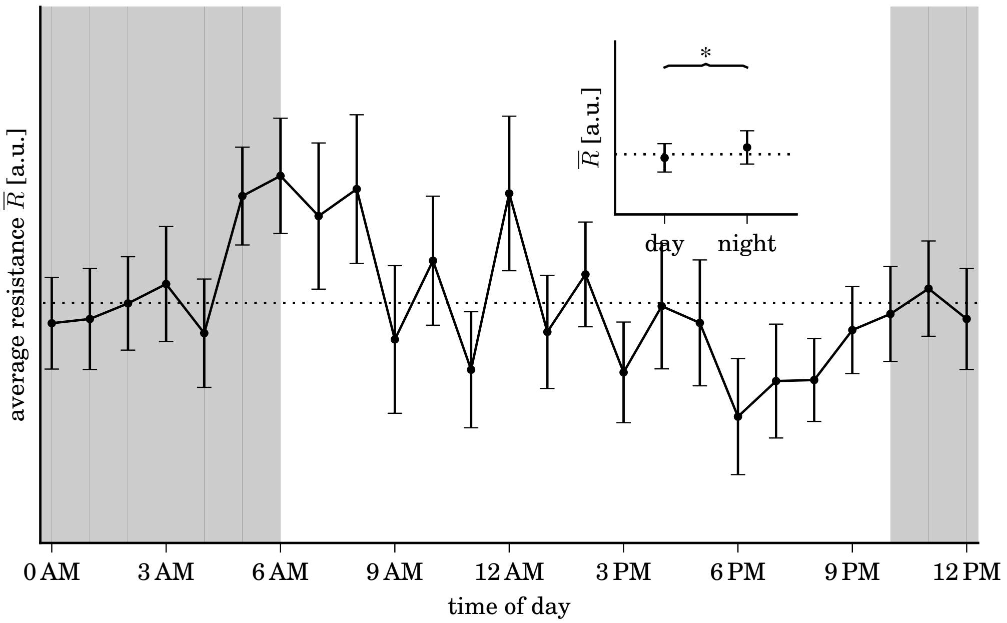

Figure S8: Same as Fig. 4 (main text), but findings derived with the information-theory-based quantifier (γ) of the dynamical coupling structure.

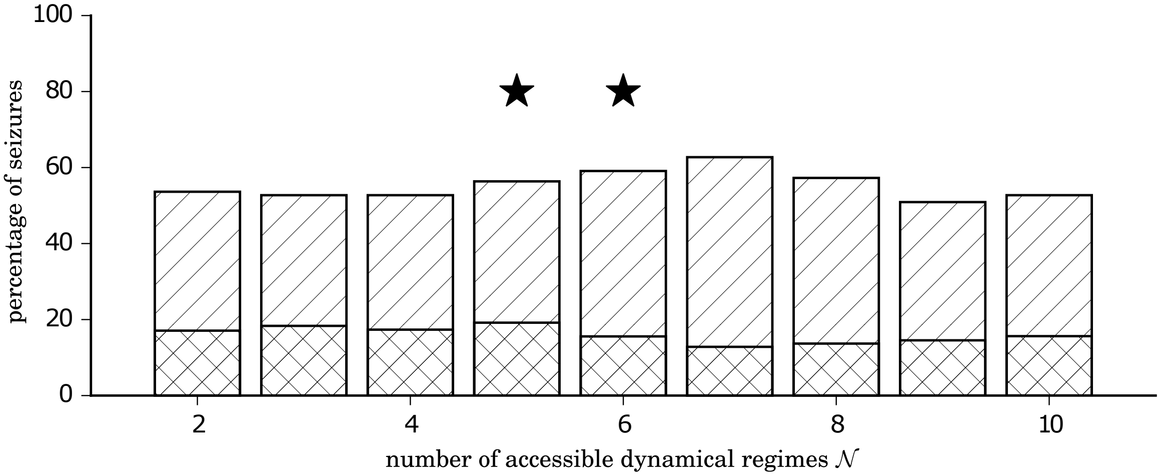

Figure S9: Same as Fig. 5b (main text), but findings derived with the information-theory-based quantifier (γ) of the dynamical coupling structure.

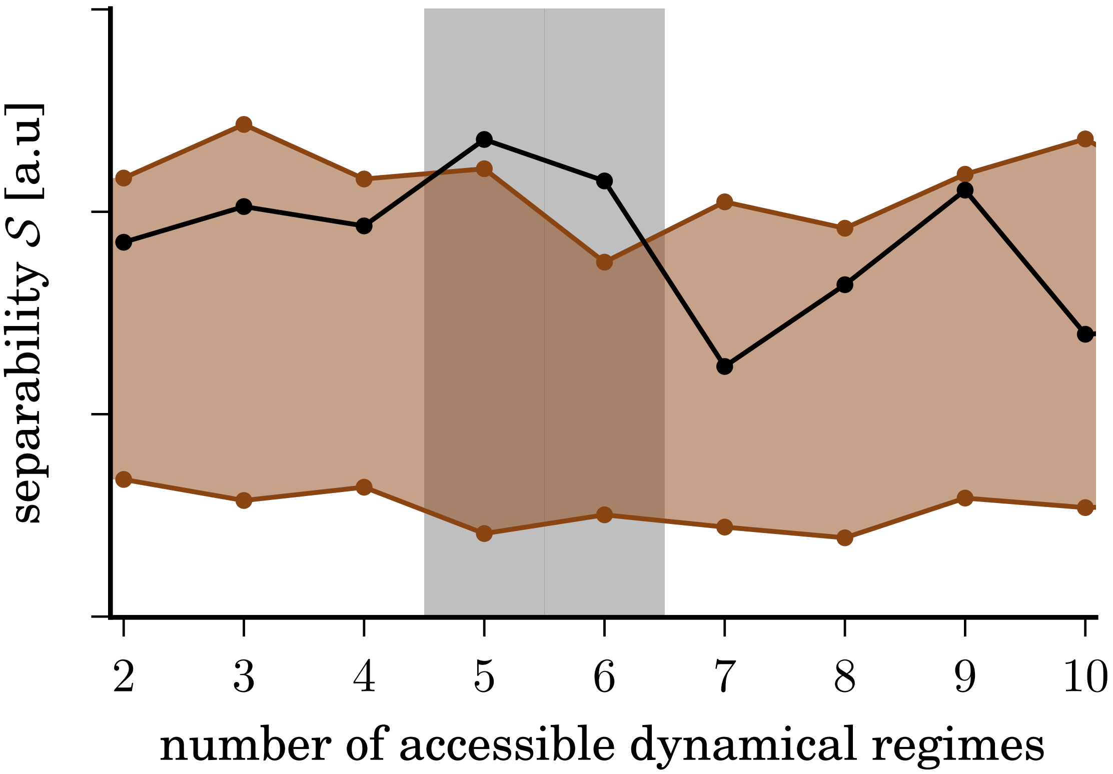

Figure S10: Same as Fig. 7b (main text), but findings derived with the information-theory-based quantifier (γ) of the dynamical coupling structure.

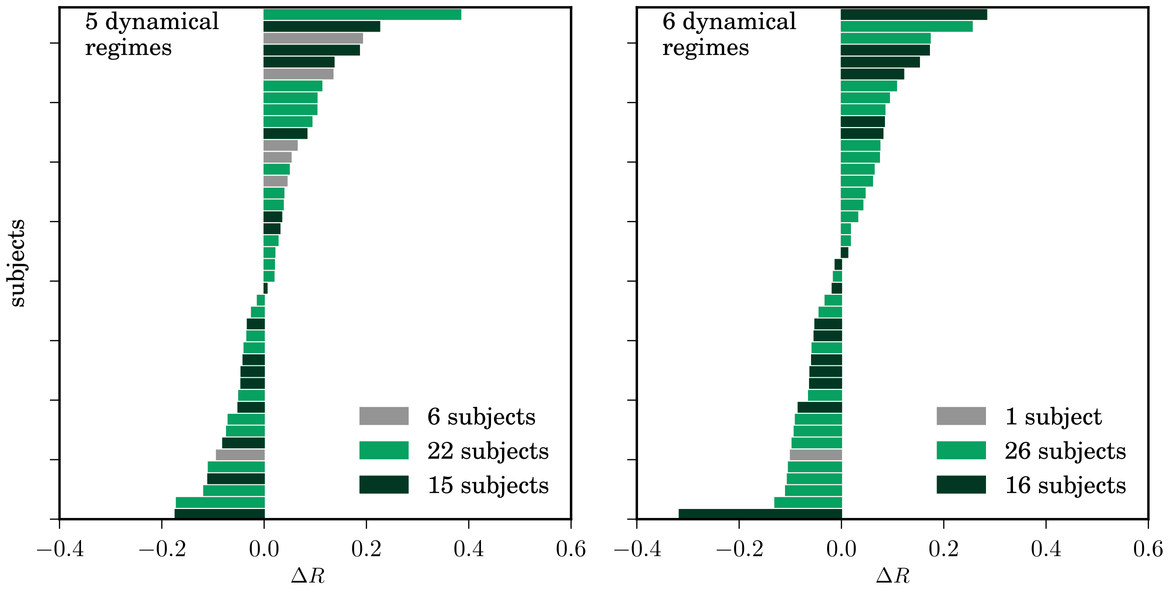

Figure S11: **Dynamical resistance of individual brains prior to epileptic seizures.** Same as Fig. S4, but findings derived with the information-theory-based quantifier (γ) of the dynamical coupling structure. Relative deviation $\Delta R = \left(\overline{R^{\text{pre}}} - \overline{R^{\text{int}}}\right) / \overline{R^{\text{int}}}$ of mean resistance values from pre-seizure periods ($\overline{R^{\text{pre}}}$) from those from inter-seizure periods ($\overline{R^{\text{int}}}$) for each subject with epilepsy and for different numbers of dynamical regimes $\mathcal{N} \in \{5, 6\}$. $\Delta R > 0$ ($\Delta R < 0$) indicates higher (lower) dynamical resistance during pre-seizure periods. The coloring of each subject's bar marks the efficiency of our resistance-monitoring approach to index precursory states: pre-seizure fluctuations outside the one-sigma (two-sigma) range of inter-seizure fluctuations are colored light green (dark green), pre-seizure fluctuations inside the distribution of inter-seizure fluctuations are colored grey.

References

1. K. Lehnertz, H. Dickten, Assessing directionality and strength of coupling through symbolic analysis: an application to epilepsy patients. *Phil. Trans. R. Soc. A* **373**, 20140094 (2015).

2. H. Dickten, S. Porz, C. E. Elger, K. Lehnertz, Weighted and directed interactions in evolving large-scale epileptic brain networks. *Sci. Rep.* **6**, 34824 (2016).

3. Z. Liu, Measuring the degree of synchronization from time series data. *Europhys. Lett.* **68**, 19–25 (2004).

4. C. Bandt, B. Pompe, Permutation entropy: A natural complexity measure for time series. *Phys. Rev. Lett.* **88**, 174102 (2002).

5. M. Staniek, K. Lehnertz, Parameter selection in permutation entropy measurements. *Int. J. Bifurcation Chaos Appl. Sci. Eng.* **17**, 3729 (2007).
